# The Effects of Regular Physical Activity and Playing Video Games on Reaction Time in Adolescents

**DOI:** 10.3390/ijerph19159278

**Published:** 2022-07-29

**Authors:** Aleksandar Klasnja, Natasa Milenovic, Sonja Lukac, Aleksandar Knezevic, Jelena Klasnja, Vedrana Karan Rakic

**Affiliations:** 1 Faculty of Medicine, University of Novi Sad, 21000 Novi Sad, Serbia; natasa.milenovic@mf.uns.ac.rs (N.M.); aleksandar.knezevic@mf.uns.ac.rs (A.K.); vedrana.karan@mf.uns.ac.rs (V.K.R.); 2 Center of Radiology, University Clinical Center of Vojvodina, 21000 Novi Sad, Serbia; sonja@lukac.rs; 3 Medical Rehabilitation Clinic, University Clinical Center of Vojvodina, 21000 Novi Sad, Serbia; 4 Blood Transfusion Institute of Vojvodina, 21000 Novi Sad, Serbia; klasnjaj@gmail.com

**Keywords:** physical activity, video games, reaction time, adolescent

## Abstract

Reaction time is of great importance in life. In both sports and video games, movements of participants are conditioned by different visual, acoustic and somatosensory signals. The aim of this research was to determine whether reaction time is influenced by regular physical activity and playing video games in adolescents. The study included 41 female and 26 male students, aged 10–14 years. Questionnaires about habits related to regular physical activity and playing video games were given to the examinees. Afterwards, the reaction time was determined for visual stimuli, via a computer program. The obtained results show that there is a statistically significant difference in the value of the reaction time of children who are regularly engaged in physical activity relative to those who play video games ((0.327 ± 0.081) s vs. (0.403 ± 0.137) s, *p* = 0.013), while there is no statistically significant difference in reaction time between children who equally participate in physical activity and video games ((0.386 ± 0.134) s) compared to those who regularly practice physical activity (*p* = 0.156), and those who only play video games (*p* = 0.610). Physical activity can decrease reaction time in children, but further studies are needed to elucidate the impact of regular physical activity and gaming on the developing adolescent brain.

## 1. Introduction

The importance as well as the beneficial effects of physical activity in adults have long been known and accepted by a large number of experts. Data on the impact of physical activity in children are less available [1,2,3,4].

Physical activity should be an integral part of growing up in young people, especially children and adolescents, because it plays a significant role in physical, social and mental development. All forms of activities, whether informal games, physical education, sports, dancing, walking, cycling, or recreational exercise, should be represented during adolescence, as they give young people valid experiences in learning basic motor skills as well as social integration, moral and social development.

Physical activity is a lifestyle that is directly and indirectly beneficial to health, primarily through preventing obesity, helping bone growth and development and joint mobility, having a beneficial effect on the cardiovascular system, promoting good mental health, and establishing healthy lifestyles which can continue into adulthood [1,4,5,6]. Participation in exercise and sports can also improve social integration, cultural tolerance, understanding of ethics and respect for the environment. It is also noteworthy that parts of the brain such as the dorsolateral prefrontal cortex, premotor cortex, and supplementary motor cortex involved in fine motor control, bimanual coordination and visuomotor skills are not fully developed until adolescence [7]. Trained motor activities are transferred and adopted in previously untrained brain areas enhancing adolescent brain maturation and plasticity [8].

Video games are part of our everyday life and are very popular among children of all ages. Modern life involves the daily use of computers, phones and tablets for various purposes. School-age children largely replace play, socializing and physical activity with a sedentary lifestyle, playing video games, and the consequences of such habits are not only reflected in poor social behavior, but also pose a risk of many locomotors, metabolic and psychiatric disorders [9,10,11]. 

A large number of papers investigating the impact of ‘gaming’ have focused on the negative aspects by studying the potential harmful effects such as aggression, addiction and depression [10,12,13]. In addition, some research shows that chronic video game play is associated with smoking, obesity, and poorer educational achievement [10,14,15]. However, there are also studies that advocate for the promotion and development of useful video games, participation in school and other educational needs in the development of children and adolescents, controlling the content of video games [16].These games may benefit motor cognitive development and have functional implications that may influence the engagement of motor systems and perceptual functions [17]. Recent studies have shown that people who play video games perceive signs better in the visual field and see more signs in total than those who do not play video games [18]. Also, surgeons who have experience in playing video games have a better and faster performance in performing laparoscopic procedures [19,20]. Enriching traditional training with innovative elements to improve motor coordination gives tangible benefits to young athletes engaged in technical sports [8].

Reaction time (RT) is a measure of the speed at which an organism responds to a stimulus. We define RT as the time required to identify a signal, which sometimes does not require conscious recognition and perception of stimuli. The duration of this time depends primarily on the type of stimulus, so we expect different results in terms of visual, somatosensory and auditory signals [21]. Factors that prolong the reaction time can be temporary and permanent. Permanent factors include: gender, personality, intelligence, education, chronic diseases of sight and hearing, while temporary factors include: type of stimulus and its intensity, drugs, psychoactive substances (alcohol as the most important), some infectious diseases, tension, fatigue. There is also a group of relatively permanent factors, the most important of which are age, physical fitness and the presence of other diseases [22].

Today, reaction time is primarily used for two purposes: to study the nature of mental processes and their basic structure by measuring the time required to perform a particular process or some of its components, and to study processes and reactions as such, by systematically manipulating stimulus and response characteristics on their execution.

RT is often overlooked and is usually considered an underestimated element in the selection of athletes for different sports. In sports games, in which the movement of the participants is conditioned by signals, movements of the opponent or the movement of the ball, RT is of great importance [23].

Previous work shows us that physical activity shortens RT in adults, in proportion to the increase in hours spent participating in any form of physical activity [24,25,26], so we expect that we will get such results in children. Due to the lack of data in the literature so far, we cannot say with certainty the positive impact of video games on RT, nor whether there is a correlation between hours spent playing video games and shortening RT. We assume that due to the large participation of the muscles of the upper extremity during playing video games, focusing and better perception of details in the field of view, RT can change. The aim of our research was to determine whether there is influence of chronic physical activity on reaction time, the impact of playing video games on reaction time and the synergistic influence of chronic physical activity and playing video games on the reaction time of school-age children.

## 2. Materials and Methods

### 2.1. Subjects

The research included students of fifth to eight grade (aged 10–14) of the local Elementary School in the village of Gospodjinci, Provice of Vojvodina, Northern Serbia. There were a total of 67 students (26 male and 41 female) who consented to participate in this study, as did their parents. The study was conducted in accordance with the Declaration of Helsinki, and the protocol was approved by the Ethics Committee of the Faculty of Medicine Novi Sad (278/18).

Each child was explained in details the method, as well as what is expected of their participation in this study. An experienced researcher was present all the time and helped complete the questionnaires with each child. All students were randomly assigned numbers 1–67 to ensure anonymity and protect the data of the subjects in this study. Excluding criteria were not signed consent forms by children’s parents.

### 2.2. Survey

Students filled out a survey in which they answered questions that were divided into two groups. The first part was a self-developed survey designed for the purposes of this study consisting of questions that showed us how long and often they played video games in the last two months, their attitudes about playing video games and how they affect their health and grades in school. The second group of questions referred to physical activity, whether they were physically active in the last two months, what sport and how long they trained, how often and for how long in the last two months they rode bicycles, ran, walked, whether they ware regular in physical education classes, etc. For this purpose we used a standardized questionnaire. The International Physical Activity Questionnaire (IPAQ) is a measurement tool that was used to obtain an objective estimate of physical activity level in the participants. The IPAQ is designed as a self-reported questionnaire for adults consisting of 27 questions that reflect on various physical activities in the past 7 days, divided into domains according to the activity types (work related, transportation, housework, recreation) [27,28]. The results can be interpreted either as categorical values (low/moderate/high level of physical activity), or as a continuous score expressed in metabolic equivalent of task (MET).

According to the data obtained from the survey, respondents were divided into four groups:

(1) Group engaged in physical activity (group S)
(2) Video game group (group V)
(3) Group engaged in physical activity and playing video games (group SV)
(4) Group that does not engage in physical activity and does not play video games (group N)

By analyzing the given answers, 27 students stated that they regularly do sports, in the last two months at least twice a week (group S), with daily performing of other physical activities such as walking, cycling, and running. These levels of activities are equivalent to moderate or high level of physical activity assessed by the International physical activity questionnaire. Twenty students stated that in the last two months they played standard video games on their computers or cell phones (available to all students included in this study) daily or more than two days a week for more than an hour a day (group V), 19 students in addition to regular training played video games (group SV), while only 1 student did not participate in physical activities as well as playing video games (group N).

### 2.3. Reaction Time Measurement

The measurement was performed with the help of a program for measuring the reaction time on a computer. We have used an online reaction time test from the author Jim Allen (Allen J. The Online Reaction Time Test. 2002, available at https://faculty.washington.edu/chudler/java/redgreen.html) (accessed on 11 July 2022) [29].

In this task, the screen shows a traffic light with 3 fields (circles): red, yellow and green; the yellow light marked the beginning, after which the respondents had to prepare their hands and press the appropriate (space) key on the keyboard. Then, by turning on the red light, the respondents were told to be on standby because turning on the green light was random, computer-determined. As soon as possible after noticing the green light, the respondents pressed the space key on the keyboard, and the first value in field 1 was shown on the screen. After this act, the yellow light came on again and the procedure was repeated.

During this task, each respondent had 20 attempts (measurements) marked with ordinal numbers 1–20. Each attempt was measured in seconds, limited to three decimal places.

The space button was pressed with the index finger of the dominant hand.

The reaction time in this case is the time that has elapsed since the green color appeared on the traffic light and the space key was pressed on the keyboard, which is measured by the computer or program itself.

### 2.4. Statistical Analysis

The Statistica 12 program (StatSoft, Tulsa, OK, USA) was used for data processing. Normal data distribution was assessed using the Kolmogorov-Smirnov test. Mean values of RV duration, standard deviation, were calculated. The existence of differences between the examined groups was done by ANOVA test and *p* values of 0.05 or lower were considered statistically significant. 

## 3. Results

The study involved 41 female students and 26 male students, aged 10–14 years. The distribution of students by sex and age are shown in Table 1.

According to the survey data, students were divided into the following groups according to the above criteria:

(1) Group engaged in physical activity (group S): 27 students
(2) Video game group (group V): 20 students
(3) Group engaged in physical activity and playing video games (group SV): 19 students
(4) Group that does not engage in physical activity and does not play video games (group N): 1 student

Due to the small sample, group N was not included in the analysis.

Analyzing the values of the reaction time, we found that there was no statistically significant difference between the values in relation to age (*p* = 0.560). Table 2 shows the mean value of RT by groups in relation to age.

Table 3 shows the values of the reaction time by groups.

ANOVA analysis showed a statistically significant difference between groups (*p* = 0.015). After the post hoc analysis, there was a difference between groups S and V (*p* = 0.013), and not between S and SV (*p* = 0.156), nor between V and SV (*p* = 0.610). 

## 4. Discussion

The results obtained in this study show that children who play video games have a prolonged reaction time compared to children who regularly engage in physical activity. Significantly lower values of RT in children who regularly play sports can be explained by the fact that chronic physical activity improves concentration and alertness, provides better muscle coordination, improves performance in speed and accuracy of tasks, reduces psychological tension and develops better contact of the mind with the body, which seems to be responsible for the better performance of the individual.

However, starting from the assumption that video games improve the motor skills of the upper part of the body, primarily by increasing the activity of the muscles of the upper extremities, the expected results of this group of students have not been met. The prolonged values of RT in group V can be explained by the fact that in this study we did not investigate the influence of different types of video games on RT.

We notice that in group SV there is an elongation of RT in relation to group S, shortening in relation to group V, but these values are not statistically significant to prove a synergistic effect.

Due to the small sample of group N that was left out of the analysis, the disadvantage of this study is that there is no control group, which would confirm the hypothesis about the influence of the mentioned variables on RT.

Hillman et al. [5] indicate that higher aerobic capacity is associated with changes in neurocognitive functions, because children with a higher degree of training showed a more efficient neuroelectric profile than children with a lower degree. Children with higher aerobic capacity have also performed better in determining response time and response accuracy, which may result from higher resource allocation to working memory, and support similar research on ability and cognition in adult populations. Despite this interesting finding, RT in children with higher aerobic capacity is still slower than in the adult group, suggesting that small but significant improvements in response rate may be associated with aerobic capacity [30]. 

The impact of regular physical activity on mental and cognitive development is almost self-explanatory. Motor and cognitive development may also be interrelated, and tasks that require parallel activation of cognitive-motor circuits encompassing the prefrontal cortex, striatum, the cerebellum may be important for cognitive functions [17]. Self-reported physical activity was associated with motor skills and academic achievement as well. Daily physical education and motor skill training during compulsory school years led to improved motor skills as well as improved school performance in adolescence [31]. It should be noted, though, that children aged 10–12 are at a different moment of their ontogenetic growth (and so are their brains) than the ones entering the puberty phase (12–13) with acceleration of stimulation and development of the brain cognitive and emotional areas. Nevertheless, we did not get any difference in time of reaction.

The association of physical activity and RT is not limited to the adolescent years, but it expands along the lifespan and similar effects as in the early adolescents in improvements of RT may be achieved also in the elderly. While fluid abilities (fluid reasoning, working memory, vision perceptual abilities, processing speed) decline with aging, crystallized intelligence (accumulated knowledge) can improve with years [32]. Physical activity and fitness level have been found to moderate these age-related changes. 

Current results indicate that there is a gender difference in RT. The two sexes differ not only biologically but also in terms of life experiences, a finding that requires additional supervision [33]. Over the past decade, opportunities have been launched to improve perceptual and cognitive abilities in video games. An insufficient amount of previous research examining how video games affect information processing, however, showed that the reaction time of children who had some experience in playing video games were significantly faster than those who did not [34].

It should be noted that this study did not evaluate the differences in motivation to participate in exercise and play video games. It is possible that this factor may play a role in the correlation of these variables with RT. Future research will need to investigate these factors further to better understand and benefit from regular physical activity and playing video games to improve RT. Also, the sample size and the study design could be limiting factors of this study. We did not control physical exercise, videogames or a combination of them for two months; this was an observational study where we recruited participants that were distinctly engaged in one of the two aforementioned activities or the combination of them. It would have also been preferable to take into consideration IQ, socioeconomic status and parental education as well.

## 5. Conclusions

The conclusion of the study is that physical activity has benefit on the time of reaction. Reaction time can be decreased in children who regularly take part in physical activity. Children playing video games have longer reaction time than children engaged in physical activity.

## Figures and Tables

**Table 1:** Distribution of the students according to age and gender.

GENDER	AGE (Years)	10	11	12	13	14	TOTAL
FEMALE		2	10	8	11	10	41
MALE		1	8	8	9	0	26
TOTAL		3	18	16	20	10	67

**Table 2:** Mean values of reaction time in relation to age.

AGE (Years)	S(s)	V(s)	SV(s)
10	0.379 ± 0.108	0.477 ± 0.112	0.495 ± 0.102
11	0.371 ± 0.121	0.443 ± 0.217	0.381 ± 0.127
12	0.324 ± 0.069	0.379 ± 0.101	0.337 ± 0.061
13	0.293 ± 0.017	0.398 ± 0.124	0.337 ± 0.116
14	0.295 ± 0.031	0.330 ± 0.015	0.428 ± 0.120

**Table 3:** Reaction time values in different groups.

GROUPS	RV(s)
S	0.327 ± 0.081 *
V	0.403 ± 0.137
SV	0.386 ± 0.134

* *p* < 0.05 between S and V groups.

## Data Availability

Not applicable.
